# Supplementary material for: Physical Quality of Life of Sepsis Survivor Severely Malnourished Children after Hospital Discharge: Findings from a Retrospective Chart Analysis
Source: Life (Basel). 2022 Mar 5;12(3):379. doi: 10.3390/life12030379 (PMC8954014; doi:10.3390/life12030379)
Supplement: Supplementary file 1 [file life-12-00379-s001.zip › life-1556367-supplementary.pdf]

Table S1. Liverpool quick Sequential Organ Failure Assessment [LqSOFA].

**Criterion**	**Points Allocated**	
	**1 Point**	**0 Points**
CRT	≥3 s	<3 s
AVPU	VPU	Alert
HR	>age-specific upper limit of normal	<age-specific upper limit of normal
RR	>age-specific upper limit of normal	<age-specific upper limit of normal

VPU= Voice, Pain, Unresponsive.

Verbal Autopsy Questionnaire

1 Was verbal autopsy being conducted?
2 The time gap between discharge and death (in days)
3 Date of the interview obtained from parents/caregiver of the deceased child
4 Visit outcome of the interview obtained from parents/caregiver of the deceased child
5 Have we been able to tell you any necessary information that you like to?
6 Do you give voluntary consent to participate?
7 Relationship of the respondent to the deceased child
8 Date of birth of the deceased child
9 Date of child's death
10 How old was the child when he/she died (in months)?
11 sex of the deceased child
12 The language used for data collection of information
13 Where did the child die?
14 Did the child have scanty micturition during the illness that led to his/her death?
15 For how long (in days) did the child have scanty micturition?
16 Did the child have red eyes during the illness that led to his/her death?
17 For how long (in days) did the child have red eyes?
18 Did the child have white patches in the tongue during the illness that led to his/her death?
19 For how long (in days) did the child have white patches in the tongue?
20 Did the child have puffiness of the face during the illness that led to his/her death?
21 For how long (in days) did the child have puffiness of the face?
22 Did the child have joint swelling during the illness that led to his/her death?
23 For how long (in days) did the child have joint swelling?
24 Did the child have leg swelling during the illness that led to his/her death?
25 For how long (in days) did the child have leg swelling?
26 Did the child have jaundice during the illness that led to his/her death?

27 For how long (in days) did the child have jaundice?

28 Did the child lose his/her weight?

29 Whether the baby was stopped feeding during an illness that led to his/her death?

30 Duration of stopped feeding (in days) during illness that led to his/her death?

31 Did the child have a cough during the illness that led to his/her death?

32 For how long (in days) did the cough continued?

33 Did the child have any breathing difficulties during the illness that led to his/her death?

34 For how long (in days) did the child have breathing difficulties?

35 Did the child have fast breathing during the illness that led to his/her death?

36 For how long (in days) did the fast breathing continued?

37 Did the child have chest indrawing during the illness that led to his/her death?

38 Did the child have grunting respiration during the illness that led to his/her death?

39 Did the child have nasal flaring during the illness that led to his/her death?

40 Did the child have any convulsion during the illness that led to his/her death?

41 Did the child have a fever during the illness that led to his/her death?

42 For how long (in days) did the fever continued?

43 Was the child's body cold by touch during the illness that led to her/his death?

44 For how long (in days) was the child's body was cold by touch?

45 Did the child become lethargic after the usual movement during the illness that led to his/her death?

46 Did the child become unconscious or not responsive to anything during the illness that led to his/her death?

47 Did the child have bulging of fontanelle during the illness that led to his/her death?

48 Did the child have any discharge of pus from the umbilicus during the illness that led to his/her death?

49 Did the child have any redness of the umbilicus during the illness that led to his/her death?

50 Did the child have any redness of the umbilicus which extended to the abdominal skin during the illness that led to his/her death?

51 Did the child have any kind of rash which occupied a large portion of the body during the illness that led to his/her death?

52 Did the child have an abscess that occupied a large portion of the body during the illness that led to his/her death?

53 Did the child have any swelling or swelling with the pus of the body during the illness that led to his/her death?

54 Did the child have bleeding from any part of the body during the illness that led to his/her death?

55 Did the child have an ulcer during the illness that led to his/her death?

56 Did the child have abnormally loose stool or loose watery stool during the illness that led to his/her death?

57 For how long (in days) did diarrhoea continued?

58 When the child suffered from severe diarrhea how many times he/she passed stool in a day (in number)

59 Whether stool was mixed with blood during the illness that led to his/her death?

60 Did the child have abdominal distension during the illness that led to his/her death?

61 For how long (in days) did the child have abdominal distension?

62 Did the abdominal distension develop rapidly?

63 Did the child have sunken eyes during the illness that led to his/her death?

64 Did the child lose skin turgor during the illness that led to his/her death?

65 Did the child vomit out all food that he/she eat during the illness that led to his/her death?

66 For how long (in days) did vomiting continued?

67 Did the child have abdominal pain during the illness that led to his/her death?

68 For how long (in days) did the child have abdominal pain?

69 Did the child have a stiff neck/painful neck during the illness that led to his/her death?

70 For how long (in days) did the child have a stiff neck/painful neck?

71 Did the child have a headache during the illness that led to his/her death?

72 For how long (in days) did the child have a headache?

73 Did the child receive any treatment for the illness that led to his/her death?

74 From where treatment was received during illness that led to his/her death?

Did you have any papers or documentation of the treatment of the child?

Table S2. Verbal autopsy to explore the condition of the deceased child before the fatal outcome.

Characteristics	Sepsis survivors n=9 (%)	Non-sepsis survivors n=15 (%)
Sex female	6 (66.7)	6 (40)
Age (months) (median, IQR)	8 (4,13)	7 (4,13)
Time gap between discharge to death (days) (median, IQR)	28 (10, 37.5)	17 (4, 24)
weight loss	9 (100)	15 (100)
Stopped feeding well	5 (55.6)	6 (40)
Cough	9 (100)	11 (73.3)
Respiratory difficulty	7 (77.8)	10 (66.7)
Fever	5 (55.6)	8 (53.3)
Loose stool	2 (22.2)	12 (80)
Abdominal distension	1 (11.1)	7 (46.7)
Vomiting	2 (22.2)	9 (60)
Thrush	1(11.1)	3 (20)
Received treatment for the illness	5 (55.6)	7 (46.7)
Visit to a qualified doctor	4/5 (80)	3/7 (42.9)
